# Domain Adaptation for In-Line Allergen Classification of Agri-Food Powders Using Near-Infrared Spectroscopy

**DOI:** 10.3390/s22197239

**Published:** 2022-09-24

**Authors:** Alexander Lewis Bowler, Samet Ozturk, Ahmed Rady, Nicholas Watson

**Affiliations:** Food, Water, Waste Research Group, Faculty of Engineering, University Park, University of Nottingham, Nottingham NG7 2RD, UK

**Keywords:** near-infrared spectroscopy, domain adaptation, transfer learning, machine learning, process monitoring, food and drink

## Abstract

The addition of incorrect agri-food powders to a production line due to human error is a large safety concern in food and drink manufacturing, owing to incorporation of allergens in the final product. This work combines near-infrared spectroscopy with machine-learning models for early detection of this problem. Specifically, domain adaptation is used to transfer models from spectra acquired under stationary conditions to moving samples, thereby minimizing the volume of labelled data required to collect on a production line. Two deep-learning domain-adaptation methodologies are used: domain-adversarial neural networks and semisupervised generative adversarial neural networks. Overall, accuracy of up to 96.0% was achieved using no labelled data from the target domain moving spectra, and up to 99.68% was achieved when incorporating a single labelled data instance for each material into model training. Using both domain-adaptation methodologies together achieved the highest prediction accuracies on average, as did combining measurements from two near-infrared spectroscopy sensors with different wavelength ranges. Ensemble methods were used to further increase model accuracy and provide quantification of model uncertainty, and a feature-permutation method was used for global interpretability of the models.

## 1. Introduction

Powdered agri-food materials are widely used in food and drink manufacturing due to their long shelf life, small volume, and ability to be easily added during processes [1]. Examples of agri-food powders include flour, coffee, dairy powders, nutritional supplements, and flavorings [1,2,3]. One problem encountered during food production is human error causing the wrong material to be added to a conveyor line. A 2019 annual report from the UK Food Standards Agency (FSA) stated that production errors, including formulation and labelling errors, accounted for 18% of product allergen incidents during the preceding year [4]. It is estimated that up to 10% of people have a food allergy in Western countries and the number is increasing [5]. Therefore, these errors can lead to product rework or waste if the final composition analysis identifies that the food contains the wrong ingredients. Furthermore, the product may have to be recalled if it has already been transported from the factory. Early detection of incorrect materials in the production line would reduce food waste and improve productivity, economics, and sustainability of agri-food systems.

Near-infrared (NIR) spectroscopy measures the absorbance of near-infrared light, which is dependent on the composition of a material. NIR is now a leading analytical tool for evaluation of food safety, authenticity, and material properties, owing to its advantages of not requiring sample preparation, being nondestructive and noninvasive, and producing real-time measurements [6]. It is also a suitable sensing technique for in-line applications for flowing solid and liquid samples derived from animal or plant sources [7,8]. NIR has been widely used to monitor properties of food powders, such as detecting adulteration [9] or determining composition [10]. Supervised machine learning (ML) techniques have been increasingly used to analyze NIR spectra, due to the overlapping peaks and nonlinear relationships between composition and absorbance intensities [11,12,13]. Supervised ML maps inputs to outputs with the aim of accurately predicting the outputs of data that were not used during training. However, in a production setting where an NIR sensor is in place to monitor a production line, collecting sufficient spectra from each material category may cause unacceptable disruption to the manufacturing process due to the time required to collect the data. ML models could be trained on static samples and directly transferred to the production line, but these are unlikely to be accurate when tested on moving materials. Several studies have shown this by focusing on the impact of motion conditions on acquired NIR spectra, for example, for monitoring minced beef [14,15], olive quality [16], food [17], and pharmaceutical powders [18,19]. For example, Dixit et al. (2016) [15] noticed increased spectral noise, and Cama-Moncunill et al. (2016) [17] showed changes to the intensity values of peaks under moving conditions. Therefore, methodologies are required to overcome the spectral shift between static and moving samples to enable effective transfer of these models. This would reduce the barriers to successful implementation of in-line NIR monitoring in industrial production lines by removing or minimizing the data-collection burden under moving conditions.

Domain adaptation is a subcategory of ML that alters how a model is trained so that it accurately predicts data from outside the range on which they were trained [20]. For this task, domain adaptation can be used to train an ML model on the spectra acquired under stationary conditions (the source domain), akin to a manufacturer collecting offline data in a laboratory, and aid transfer of these models to the moving conditions on a conveyor line (the task domain). Matrix or kernel-based methods have previously been used for domain adaptation of 1D NIR spectra to find projections between domains [21,22] or to also extract discriminative features to be used for a main learning task [23,24,25,26]. Ensemble methods that maximize model diversity and prediction similarity in the new domain data have also been used to increase the probability that transferable correlations are used [27]. However, deep-learning methods based on neural networks are common when using domain adaptation on visible-light images [28] or 2D NIR images [29,30], particularly domain-adversarial neural networks [31,32]. In this work, two deep-learning domain-adaptation approaches are investigated: semisupervised generative adversarial networks (SGANs) and domain-adversarial neural networks (DANNs). These methods are used both individually and combined and compared to using transfer learning without domain adaptation. Using these methods with no labelled data from the target domain is also compared to using a single labelled instance from each material category collected under moving conditions. To the authors’ knowledge, this is the first use of deep learning for 1D NIR spectral domain adaptation and the first use of SGANs with NIR spectra. Two NIR sensors covering different wavelength ranges and three motion speeds are investigated. The ML task aims to classify five allergen groups for 19 food-powder materials. The methods are able to reduce the barrier to successful implementation of NIR and ML combinations in production environments by minimizing the data-collection burden and enhancing trust in the models’ predictions.

## 2. Materials and Methods

The aim of the classification task was to identify the allergen category to which each food powder material belonged. The five allergen classes were: gluten, gluten-free, peanut, tree nut, and egg (Table 1). A total of 19 food-powder materials were investigated. The materials were purchased from stores in Nottingham, UK.

### 2.1. Near-Infrared Spectroscopy Measurement

**Figure 1:**
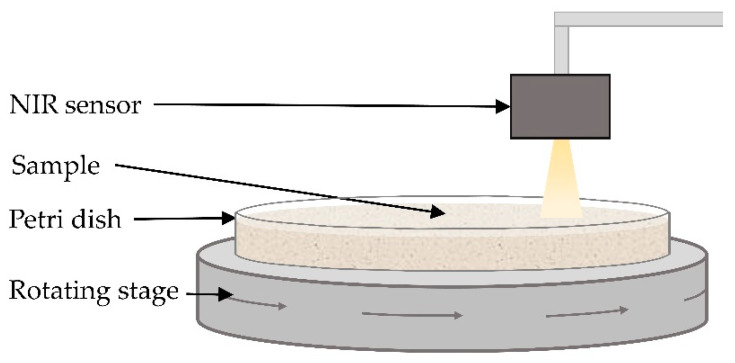
A diagram of the experimental setup. Two NIR sensors were used (S2.0 and S2.5). The distance between the NIR sensors and the sample surface was 2 cm. Samples weighing 100 g were measured in a 12 cm-diameter petri dish. The samples were measured under stationary conditions and at three speeds (slow—0.017 ms^−1^, medium—0.036 ms^−1^, fast—0.068 ms^−1^) using the rotating stage.

All materials were measured using two NIR sensors: NIRONE S2.0 (wavelength range: 1550 to 1950 nm, 1 nm resolution) and S2.5 (wavelength range: 2000 to 2450 nm, 1 nm resolution) (Spectral Engines, Oulu, Finland). Before acquisition of each spectrum, a white reference spectrum at 90% light intensity and a dark background spectrum at zero light intensity were collected. The distance between the NIR sensors and the sample surface was maintained at 2 cm for all measurements. Samples weighing approximately 100 g were placed in a 12 cm-diameter glass petri dish and compressed to obtain a flat surface (Figure 1). The samples were measured under static and motion conditions at three speeds: 0.017, 0.036, and 0.068 ms^−1^ (designated as slow, medium, and fast throughout, respectively). For the static measurements, between each spectrum acquisition, the petri dish was rotated so that spectra were collected from all areas of the sample. For the measurements under motion conditions, the petri dishes were placed on a rotational sample holder that had variable speed settings. Five samples were measured from each material and ten spectra were acquired from each sample, producing a total of 50 spectra for each material and for each sensor. For the materials sourced from three brands (see Table 1), 3 × 50 spectra for each material and each sensor were collected. Therefore, in total, 1250 spectra were collected using each sensor at each speed (stationary, slow, medium, and fast).

### 2.2. Domain Adaptation

ML models correlate inputs with outputs (also called labelled data) during training. When the distribution of the input data changes, erroneous predictions can be obtained. Domain adaptation is a category of ML technique that alters how a model is trained or predicts to enable it to accurately predict on data outside its training set distribution. In this work, the aim is to transfer ML models trained on NIR spectra acquired under stationary conditions to accurately predict under moving conditions with none or few labelled data. This would enable NIR and ML combination deployment in industrial environments by either negating or reducing the data to collect under moving conditions and thereby minimizing disruption to a manufacturing process. Two domain-adaptation techniques were investigated: DANNs and SGANs. Both methods were investigated individually and combined. These models were compared with transfer learning using no domain adaptation. The methods were investigated using no labelled data from the target domain or using a single labelled instance from each material category in the target domain.

#### 2.2.1. Domain-Adversarial Neural Networks

**Figure 2:**
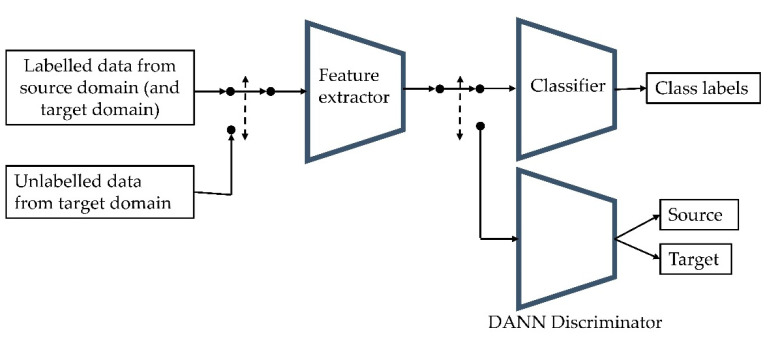
A diagram of the domain-adversarial neural network (DANN) structure and training procedure. The network was trained in three steps, iteratively: (1) the feature extractor and classifier were trained to classify the labelled data, (2) the discriminator module was trained to classify the domain of the labelled data and the feature extractor was trained to confuse the discriminator, (3) the discriminator module was trained to classify the domain of the unlabelled data and the feature extractor was trained to confuse the discriminator.

DANNs are trained using labelled data from the source domain (or also from the target domain) and unlabeled data from the target domain (Figure 2). The aim is to extract features discriminative as to the class of allergen but nondiscriminative as to which domain the data are taken from. This is achieved through training the classifier module whilst simultaneously confusing the discriminator module as to whether the input data are from the source or target domain [33]. The network was trained on three losses sequentially. Firstly, the feature extractor and classifier are trained to accurately classify the labelled data. Secondly, the discriminator module is trained to accurately classify whether the labelled data are from the source or target domain. As it is desired that the feature extractor learns nondiscriminative features between the domains, it is trained on the negative inverse of this loss to encourage gradient ascent, i.e., if the discriminator is highly accurate, a large step is taken to move the feature-extractor weights away from local optima. If the discriminator has low accuracy, a smaller gradient ascent step is taken with the feature-extractor weights. Thirdly, in a similar way to previously described, the discriminator is then trained to determine whether the unlabeled data are from the target domain and the feature extractor is trained on the negative inverse of this loss.

#### 2.2.2. Semisupervised Generative Adversarial Neural Networks

**Figure 3:**
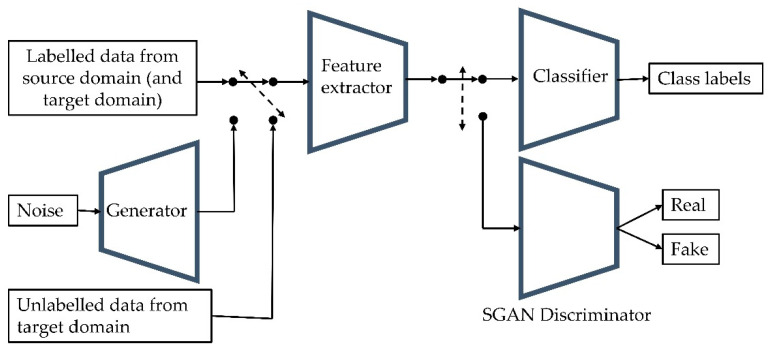
A diagram of the semisupervised generative adversarial network (SGAN) structure and training procedure. The network was trained in three steps, iteratively: (1) the feature extractor and discriminator were trained to classify that the labelled data were real, (2) the feature extractor and discriminator were trained to classify that the generated samples were fake and the generator was trained to confuse the discriminator, (3) the feature extractor and discriminator were trained to classify that the unlabelled target domain data were real.

SGANs, in conjunction with using labelled data for the main learning task, train a generator to generate fake data samples and a discriminator to classify whether each input data sample is real or fake [34]. In this work, the generator was trained to produce spectra similar to the target domain data. This encourages the feature extractor to learn discriminative features to classify between allergen types whilst also being discriminative to the real target domain data compared to the generated samples. The SGAN was trained on three losses sequentially (Figure 3). Firstly, the feature extractor and discriminator were trained to determine that the labelled data were real. Secondly, the feature extractor and discriminator were trained to determine that the generated samples were fake. The generator was trained on the negative inverse of this loss to encourage gradient ascent away from the local minima. Lastly, the feature extractor and discriminator were trained to predict that the unlabelled target domain data were real.

#### 2.2.3. Model Training

The models were trained using a batch size of 32, a learning rate of 0.001, and the Adam optimization algorithm for 10,000 epochs. The feature-extractor module consisted of four fully connected layers with 256, 128, 64, and 32 neurons, respectively. The classifier module consisted of a single logistic regression layer connecting the outputs of the last feature-extractor layer to the five allergen classes. The generator module consisted of five fully connected layers with 32, 64, 128, 256 neurons, and finally the size of the spectra being generated, respectively. Both discriminator modules for the DANN and SGAN networks consisted of a single logistic regression layer connecting the last layer of the feature extractor to the predicted classes: whether the input data were sampled from the source or target domain for the DANN discriminator, or whether the input data were real or generated for the SGAN discriminator. The hyperparameters were chosen by achieving 100% accuracy on the training set.

The models were trained using labelled data from all spectra acquired under static conditions, i.e., 50 stationary spectra for each material (or material brand). This totals 1250 spectra for each sensor used. Either none or a single labelled instance of each material category collected under moving conditions was also added to the training set. Half of the spectra (625 spectra per sensor, 25 spectra per material) for each material or material brand collected under moving conditions was used as an unlabelled dataset during training. The remaining moving condition spectra (625 spectra per sensor, 25 spectra per material) were used as the test set.

#### 2.2.4. Trust in Machine Learning

Trust in model predictions is required to facilitate acceptance of ML models in manufacturing environments and for operators to make decisions based on their outputs. Three key components of trust are accuracy, uncertainty quantification, and interpretability. Accuracy is commonly reported and refers to the proportion of correct predictions an ML model makes. Uncertainty quantification requires an estimate of how confident the model is in its prediction. Uncertainty estimates can provide information as to whether a model’s prediction should be trusted or whether further investigation is necessary. Interpretability necessitates information about how the model is deciding its predictions.

In this work, the prediction accuracy is reported as the percentage of correctly classified allergen categories for the test set acquired under moving conditions. For uncertainty quantification, an ensemble of five deep neural networks were trained, taking advantage of random initialization to produce models with different final weights. Ensemble learning techniques are one of the widest used uncertainty-quantification techniques [35]. Ensemble methods are usually used to combine predictions from multiple ML models to increase prediction accuracy, but they can also provide an uncertainty estimation by reporting the level of consensus among the individual models. To interpret the trained models, a permutation-based method was used to determine the most important wavelengths to the prediction made by the ensemble model. This is a global feature-importance method that randomly shuffles each feature (in this case, the intensity values of each wavelength) and measures the reduction in accuracy caused by this augmentation [36]. The augmented wavelengths that cause the largest reduction in accuracy are most important to the model’s prediction.

## 3. Results

The model prediction-accuracy results are presented in Table 2. Overall, high accuracy were obtained through using none (up to 96%) or a single instance (up to 99.68%) of labelled data from each material under moving conditions. This demonstrates that the domain-adaptation methodology is suitable for transfer across stationary and moving samples. Therefore, the methodology has the potential to minimize disruption to a food manufacturing process by reducing the burden of collecting labelled data under moving conditions. These results were more accurate than using transfer learning alone (i.e., not using the DANN or SGAN domain-adaptation methodology), which achieved up to 92.96% with no labelled data or 96.8% using a single instance of labelled data from each material under moving conditions. This demonstrates that the domain-adaptation methodologies improved the extraction of features that were applicable to the spectra acquired under moving conditions.

In isolation, the DANN approach achieved higher accuracy than using the SGAN training for four out of six tasks and achieved higher accuracy than using both combined for two out of six tasks. However, the SGAN approach still achieved higher accuracy than the transfer learning approach for five out of six tasks, proving that it was useful in extracting discriminative target domain features. Using the DANN and SGAN approaches together achieved the highest prediction accuracy for four out of six tasks compared with using either method alone. This indicates that the domain nondiscriminative features learned by the DANN are enhanced by the target domain discriminative features extracted through the SGAN training.

**Figure 4:**
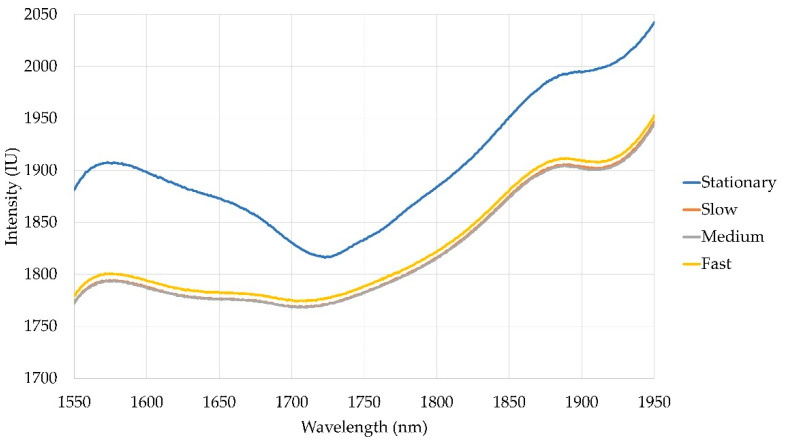
Plots of the average spectra for barley flour under stationary and each moving condition (slow—0.017 ms^−1^, medium—0.036 ms^−1^, fast—0.068 ms^−1^).

Interestingly, for models using no labelled data from the target domain, an increase in accuracy was found with increasing speed. Furthermore, for models using a single labelled instance from each material, the highest accuracy was achieved at the medium-speed setting. This is counterintuitive, as higher accuracy was expected at the lowest speeds due to the slower moving materials causing less distribution in intensity values (Figure 4) [15]. However, it is possible that at higher speeds, the larger intensity distribution enables the networks to better adapt to samples at the edges of the distribution and encourages learning of features based on spectra shape, rather than average intensity values.

Using both sensors achieved the highest prediction accuracy, with the S2.0 sensor alone producing more accurate predictions than the S2.5 sensor. This indicates that the more important wavelength ranges for discriminating between materials are located in the S2.0 wavelength range, but for accurate monitoring of a conveyor line, installation of both sensors is optimal. Furthermore, using a single labelled instance for each material category under moving conditions improved model accuracy over using no labelled data from the target domain (e.g., from 95.04% to 99.36% using the SGAN and DANN methods combined for the slow speed). Therefore, provided the level of process disruption is acceptable, a small number of labelled samples under moving conditions should be collected on the conveyor system to improve model accuracy.

### 3.1. Uncertainty

To illustrate how these models could include uncertainty as a trust measure in an industrial setting, an ensemble of five neural networks was trained, providing a distribution of predictions owing to the random initialization of the network weights. This was undertaken using the combined DANN and SGAN model using no labelled samples in the target domain, to predict for samples under the fast-moving conditions and using data from both sensors. This sensing combination (SGAN and DANN, both sensors) was selected as it produced overall the highest model accuracy and this task combination (fast speed, no moving condition labelled data) was chosen as it represents the most difficult task. Overall, the ensemble of models achieved an accuracy of 96.96% (Table 3) on the moving condition samples compared with 95.04% achieved by a single model alone, as reported in Table 2. This illustrates the improved model accuracy achievable by ensemble methods whilst also providing a quantification of model confidence. The confidence scores and corresponding model performance are presented in Table 3. A confidence score of 0.6 indicates that three out of five models predicted that class, whereas a confidence score of 1.0 indicates that all models predicted the same class.

Notably, as the confidence score increases, the percentage of incorrectly classified samples with this confidence score decreases (31.8 to 1.4%, Table 3). This demonstrates that the ensemble method is less confident with incorrectly predicted materials. This validates the proposed method’s efficacy in quantifying model uncertainty and subsequently enhancing model trust. In an industrial environment, a threshold value could be implemented based on the model’s confidence in its prediction. Therefore, if in addition to predicting the material on the conveyor, the model also presents its confidence, production would only be stopped if the prediction confidence score were above this threshold. Importantly, there were eight samples where all the models incorrectly predicted the material. Therefore, it is likely that a greater number of networks in the ensemble is required. In an industrial environment during production, if it were found that confident incorrect predictions were being made, additional networks could be trained to form part of the ensemble.

A detailed breakdown of the incorrectly identified samples is presented in Table 4. Gluten-free and peanut allergen categories produced the most false negatives, with a total of seven instances each. In particular, coconut flour produced five false-negative predictions with oat flour and peanut flour producing four each. All of these instances were predicted as gluten-containing materials. The gluten allergen category produced the most false-positive predictions, with a total of 15 instances. However, the most frequent high-confidence false positives were peanut-containing products being classified as gluten-containing. This suggests that rather than adding additional networks uniformly to all material classes, this could be tailored based on observed misclassifications. For example, a greater number of networks can be trained to only be used when a production line that is meant to contain peanut-based products is predicted as containing gluten. Another solution may be to implement higher confidence limits for these scenarios.

### 3.2. Interpretability

Important wavelength ranges were determined through permutation of individual wavelength values and by monitoring the reduction in prediction accuracy. From this, five wavelength ranges were identified (Table 5). Interestingly, the most important wavelength range (1785–1870 nm) did not correspond to a presence of powder components. However, the second (1686–1744 nm) and third (1931–1951 nm) most important wavelength ranges did correspond to the presence of long-chain fatty acids and water absorption, respectively. This indicates that the most important wavelength range was identified as it held information about both of the other two wavelength ranges that were deemed less important when used on their own. The fourth most important wavelength range (2111–2132 nm) indicated a protein-absorption band, and the fifth most important wavelength range (1569–1604 nm) corresponded to a carbohydrate-absorption band. Most of the important wavelength ranges were found within the S2.0 sensor wavelength range (1550–2000 nm), explaining why the S2.0 sensor achieved higher prediction accuracy than the S2.5 sensor. However, despite this, better accuracy was observed using both sensors together, suggesting that there is still useful information to be extracted by incorporating the S2.5 sensor in the prediction task.

### 3.3. Comparison to Previous Work

The results in this work are in close agreement with those presented in the literature that have also compared domain-adaptation methodologies to transfer learning alone using NIR spectroscopy. In summary of this work, an increase in prediction accuracy of up to 7.84% was achieved through using the DANN and SGAN methodologies combined compared with transfer learning alone (Table 2, medium speed, no labelled data instances). Similarly, Zhang et al. (2022) [29] used domain adaptation to allow the use of visible-light image datasets to train networks for infrared pedestrian detection. Domain adaptation was used to align the features between the infrared and visible-light domains. A domain classifier and gradient reversal layer were used to achieve this, a method similar to the DANNs used in this work. The feature-extraction module was updated in the direction of increasing domain classification loss to enable alignment of the feature space from both domains. Compared with transfer learning alone, using domain adaptation with the EfficientDet network increased the average precision by 2.0% on the XDU-NIR2020 dataset and 2.2% on the CVC-09 dataset. Mishra and Nikzad-Langerodi (2020) [42] and Mishra et al. (2020) [43] compared partial least squares regression and domain-invariant partial least squares regression (di-PLS), and dynamic orthogonal projections and transfer component analysis (TCA), respectively. Di-PLS uses a regularization term to minimize the variability between both domains whilst maximizing covariance between the source domain and response variables. This is followed by ordinary PLS, where latent variables that explain most of the variability in the data are extracted. TCA aims to minimize the distance between the source and target domains whilst maximizing variance in the data. A disadvantage of these methods compared to the deep-learning approaches used in this work is their limited feature-extraction capability. When using deep neural networks, not only can features from the original spectra be amplified throughout the network layers, but relationships between wavelength intensities can also be considered [12]. Both works used these methods for analysis of fresh fruit samples. The domain-adaptation approaches were used to overcome spectral changes due to using different instruments, different operating temperatures, or seasonal variations for the fruit samples. Mishra and Nikzad-Langerodi (2020) [42] achieved increases in R^2^ by up to 67% and decreases in prediction bias and root mean squared error (RMSE). Similarly, Mishra et al. (2020) [43] noticed increases in R^2^ of up to 31% and 98% and 66% reductions in prediction bias and RMSE, respectively. This similarity to other works indicates that the methods presented in this work are suitable for improving in-line allergen detection and minimizing the data-collection burden in industrial environments.

## 4. Conclusions

A common problem in food manufacturing is human error causing the wrong powder material being loaded onto a production line. In-line NIR combined with ML is a solution to enable early detection of this problem. However, a method is needed to minimize the data-collection burden when deploying this solution in manufacturing environments to minimize process disruption. This work investigated two deep-learning domain-adaptation methodologies (DANNs and SGANs) to transfer ML models trained using spectra acquired under stationary conditions, akin to collecting spectra in a laboratory, to accurately predict using spectra acquired whilst moving, i.e., when the sensor is implemented above a conveyor. Combining both methods worked best, as did combining spectra from two NIR sensors with different wavelength ranges. Overall, accuracy of up to 96.0% was achieved using no labelled instances from the moving target domain data and up to 99.68% when incorporating a single labelled instance for each material category. The use of ensemble methods was shown to increase accuracy and provide a measure of model prediction confidence, and a feature-permutation method was used for global interpretability of the models. The most high-confidence false positives were produced when peanut-containing materials were incorrectly classified as containing gluten. This indicates that a greater number of neural networks in the ensemble models could be used for these cases or a higher confidence threshold could be utilized. Implementation of this screening method in production lines could help to reduce food waste and improve productivity, economics, and sustainability of agri-food systems.

## Figures and Tables

**Table 1:** Food powder materials measured using near-infrared spectroscopy. In total, 19 food powder materials were measured.

Allergen	Materials
Gluten	Spelt, rye, buckwheat, oat, barley, brown, wheat (three brands), and wheat gluten flours
Gluten-free	Gluten-free white (three brands), coconut, tapioca, corn, and rice flours
Peanut	Peanut and peanut butter powders
Tree nut	Almond flour (three brands)
Egg	Whole egg, egg yolk, and egg white powders

**Table 2:** The accuracies (%) for each domain-adaptation methodology on the test set acquired under moving conditions for each speed. A comparison between using both sensors and each sensor individually is provided. A comparison to using transfer learning (TL) with no domain-adaptation methodology is included. None or single in the labelled data instances row indicates whether no labelled data from the spectra acquired under moving conditions was used in the training set or if a single labelled instance from each material was included. The bolded results show the highest accuracies achieved for each task (i.e., combination of speed and number of labelled target domain data instances).

		Accuracy (%)							
Method		SGAN + DANN		SGAN		DANN		TL	
Labelled Data Instances		None	Single	None	Single	None	Single	None	Single
Sensor	Speed								
Both	Slow	**95.04**	**99.36**	89.76	98.56	94.08	99.04	89.92	96.80
	Medium	**95.20**	99.20	92.48	95.70	92.16	**99.68**	87.36	94.72
	Fast	95.04	**98.24**	93.76	97.44	**96.00**	97.12	92.96	94.08
S2.0	Slow	93.20	95.60	94.12	92.88	92.76	95.00	88.56	93.34
	Medium	91.60	92.12	90.80	94.32	89.72	96.72	88.32	92.96
	Fast	86.36	91.64	87.40	90.12	87.80	88.28	86.56	89.12
S2.5	Slow	60.96	89.12	65.28	84.32	79.36	80.80	70.40	90.88
	Medium	65.12	84.16	65.60	84.32	72.00	82.88	61.12	82.88
	Fast	65.12	86.56	74.72	77.92	74.08	87.84	71.20	86.88

**Table 3:** A summary of the accuracy and confidence of the ensemble model predictions on the test set data. The ensemble model consisted of five networks. As a case study, the networks were trained using both SGAN and DANN methods and both sensor measurements to predict for the fast speed with no labelled data from the target domain. A confidence score of 0.6 indicates that three out of five models predicted that class, whereas a confidence score of 1.0 indicates that all models predicted the same class.

	Confidence Score						
	0.6		0.8		1.0		Total Count
	Count	Percentage	Count	Percentage	Count	Percentage	
Correct	15	68.2	38	90.5	553	98.6	606
Incorrect	7	31.8	4	9.5	8	1.4	19
Total	22		42		561		625

**Table 4:** A breakdown of the incorrectly predicted allergen classes for the ensemble model. The ensemble model consisted of five networks. As a case study, the networks were trained using both SGAN and DANN methods and both sensor measurements to predict for the fast speed with no labelled data from the target domain.

Misclassified Materials	Real Allergen Category	Predicted Allergen Category	Frequency (Confidence Scores)
Coconut flour	Gluten-free	Gluten	5 (0.8, 0.6, 0.6, 0.6, 0.6)
Egg yolk powder	Egg	Gluten	1 (0.6)
Gluten free white flour	Gluten-free	Gluten	1 (0.6)
Oat flour	Gluten	Peanut	4 (1.0, 1.0, 0.8, 0.6)
Peanut flour	Peanut	Gluten	4 (1.0, 1.0, 1.0, 0.8)
Peanut butter powder	Peanut	Gluten	3 (1.0, 1.0, 1.0)
Rice flour	Gluten-free	Gluten	1 (0.8)

**Table 5:** Important wavelength ranges for the ensemble model and spectral features located in these ranges. The wavelength ranges are listed in order of importance from top to bottom.

Wavelength Range (nm) (in Order of Importance, Top to Bottom)	Spectral Features
1785–1870	Range between long-chain fatty acid and water-absorption bands
1686–1744	Long-chain fatty acids producing a CH2 first overtone at 1725–1750 nm [37,38]
1931–1951	Water-absorption bands due to the vibration of O-H bonds [39]
2111–2132	Protein-absorption band [37,40,41]
1569–1604	O-H stretching of the first overtone in carbohydrates [17]

## Data Availability

The researchers at the University of Nottingham can be contacted for access to data.

## References

[1] Gao W., Chen F., Wang X., Meng Q. (2020). Recent advances in processing food powders by using superfine grinding techniques: A review. Compr. Rev. Food Sci. Food.

[2] Okere E.E., Arendse E., Nieuwoudt H., Fawole O.A., Perold W.J., Opara U.L. (2021). Non-invasive methods for predicting the quality of processed horticultural food products, with emphasis on dried powders, juices and oils: A review. Foods.

[3] Juarez-Enriquez E., Olivas G.I., Zamudio-Flores P.B., Perez-Vega S., Salmeron I., Ortega-Rivas E., Sepulveda D.R. (2022). A review on the influence of water on food powder flowability. J. Food Process. Eng..

[4] Food Standard Agency Business Committee Meeting—September 2020. https://www.food.gov.uk/sites/default/files/media/document/fsa-20-09-12-incidents-resilience-annual-report.pdf.

[5] Loh W., Tang M.L.K. (2018). The epidemiology of food allergy in the global context. Int. J. Environ. Res. Public Health.

[6] Qu J.-H., Liu D., Cheng J.-H., Sun D.-W., Ma J., Pu H., Zeng X.-A. (2015). Applications of Near-infrared Spectroscopy in Food Safety Evaluation and Control: A Review of Recent Research Advances. Crit. Rev. Food Sci. Nutr..

[7] Wang L., Sun D.-W., Pu H., Cheng J.-H. (2017). Quality analysis, classification, and authentication of liquid foods by near-infrared spectroscopy: A review of recent research developments. Crit. Rev. Food Sci. Nutr..

[8] Porep J.U., Kammerer D.R., Carle R. (2015). On-line application of near infrared (NIR) spectroscopy in food production. Trends Food Sci. Technol..

[9] Ma H.-L., Wang J.-W., Chen Y.-J., Cheng J.-L., Lai Z.-T. (2017). Rapid authentication of starch adulterations in ultrafine granular powder of Shanyao by near-infrared spectroscopy coupled with chemometric methods. Food Chem..

[10] Ingle P.D., Christian R., Purohit P., Zarraga V., Handley E., Freel K., Abdo S. (2016). Determination of protein content by NIR spectroscopy in protein powder mix products. J. AOAC Int..

[11] Morellos A., Pantazi X., Moshou D., Alexandridis T., Whetton R., Tziotzios G., Wiebensohn J., Ralf B., Mouazen A.M. (2016). Machine learning based prediction of soil total nitrogen, organic carbon and moisture content by using VIS-NIR spectroscopy. Biosyst. Eng..

[12] Ng W., Minasny B., Montazerolghaem M., Padarian J., Ferguson R., Bailey S., McBratney A.B. (2019). Convolutional neural network for simultaneous prediction of several soil properties using visible/near-infrared, mid-infrared, and their combined spectra. Geoderma.

[13] Chen H., Chen A., Xu L., Xie H., Qiao H., Lin Q., Cai K. (2020). A deep learning CNN architecture applied in smart near-infrared analysis of water pollution for agricultural irrigation resources. Agric. Water Manag..

[14] Hildrum K.I., Nilsen B.N., Westad F., Wahlgren N.M. (2004). In-line analysis of ground beef using a diode array near infrared instrument on a conveyor belt. J. Near Infrared Spectrosc..

[15] Dixit Y., Casado-Gavalda M.P., Cama-Moncunill R., Cama-Moncunill X., Cullen P.J., Sullivan C. (2016). Prediction of beef fat content simultaneously under static and motion conditions using near infrared spectroscopy. J. Near Infrared Spectrosc..

[16] Salguero-Chaparro L., Baeten V., Fernández-Pierna J.A., Peña-Rodríguez F. (2013). Near infrared spectroscopy (NIRS) for on-line determination of quality parameters in intact olives. Food Chem..

[17] Cama-Moncunill R., Markiewicz-Keszycka M., Dixit Y., Cama-Moncunill X., Casado-Gavalda M.P., Cullen P.J., Sullivan C. (2016). Multipoint NIR spectroscopy for gross composition analysis of powdered infant formula under various motion conditions. Talanta.

[18] Berntsson O., Danielsson L.-G., Folestad S. (2001). Characterization of diffuse reflectance fiber probe sampling on moving solids using a Fourier transform near-infrared spectrometer. Anal. Chim. Acta.

[19] Martínez L., Peinado A., Liesum L., Betz G. (2013). Use of near-infrared spectroscopy to quantify drug content on a continuous blending process: Influence of mass flow and rotation speed variations. Eur. J. Pharm. Biopharm..

[20] Bowler A.L., Pound M.P., Watson N.J. (2022). A review of ultrasonic sensing and machine learning methods to monitor industrial processes. Ultrasonics.

[21] Andries E., Kalivas J.H., Gurung A. (2019). Sample and feature augmentation strategies for calibration updating. J. Chemom..

[22] Lin J., Zhang L., Li J., Zhou W. A feature domain space transfer method for improving identification of maize haploid seed based on near-infrared spectroscopy. Proceedings of the 2019 International Conference on High Performance Big Data and Intelligent Systems (HPBD&IS).

[23] Huang G., Chen X., Li L., Chen X., Yuan L., Shi W. (2020). Domain adaptive partial least squares regression. Chemom. Intell. Lab. Syst..

[24] Mishra P., Nikzad-Langerodi R. (2021). A brief note on application of domain-invariant PLS for adapting near-infrared spectroscopy calibrations between different physical forms of samples. Talanta.

[25] Shi G., Cao J., Li C., Liang Y. (2020). Compression strength prediction of Xylosma racemosum using a transfer learning system based on near-infrared spectral data. J. For. Res..

[26] Diaz V.F., Mishra P., Roger J.-M., Saeys W. (2022). Domain invariant covariate selection (Di-CovSel) for selecting generalized features across domains. Chemom. Intell. Lab. Syst..

[27] Spiers R.C., Kalivas J.H. (2021). Reliable Model Selection without Reference Values by Utilizing Model Diversity with Prediction Similarity. J. Chem Inf Model..

[28] Zhuang F., Qi Z., Duan K., Xi D., Zhu Y., Zhu H., Xiong H., He Q. (2021). A Comprehensive Survey on Transfer Learning. Proc. IEEE.

[29] Zhang J., Liu C., Wang B., Chen C., He J., Zhou Y., Li J. (2022). An infrared pedestrian detection method based on segmentation and domain adaptation learning. Comput. Electr. Eng..

[30] Nikisins O., George A., Marcel S. Domain Adaptation in Multi-Channel Autoencoder based Features for Robust Face Anti-Spoofing. Proceedings of the 2019 International Conference on Biometrics (ICB).

[31] Song S., Yu H., Miao Z., Fang J., Zheng K., Ma C., Wang S. (2020). Multi-spectral salient object detection by adversarial domain adaptation. Proc. AAAI Conf. Artif. Intell..

[32] Song S., Miao Z., Yu H., Fang J., Zheng K., Ma C., Wang S. (2022). Deep Domain Adaptation Based Multi-Spectral Salient Object Detection. IEEE Trans. Multimed..

[33] Ganin Y., Ustinova E., Ajakan H., Germain P., Larochelle H., Laviolette F., Marchand M., Lempitsky V. (2016). Domain-adversarial training of neural networks. J. Mach. Learn. Res..

[34] Salimans T., Goodfellow I., Zaremba W., Cheung V., Radford A., Chen X. Improved techniques for training GANs. Proceedings of the Advances in Neural Information Processing Systems 29: Annual Conference on Neural Information Processing Systems 2016.

[35] Abdar M., Pourpanah F., Hussain S., Rezazadegan D., Liu L., Ghavamzadeh M., Fieguth P., Cao X., Khosravi A., Acharya U.R. (2021). A review of uncertainty quantification in deep learning: Techniques, applications and challenges. Inf. Fusion.

[36] Molnar C. (2022). Interpretable Machine Learning: A Guide for Making Black Box Models Explainable (2nd ed.). Christophm.github.io/interpretable-ml-book/.

[37] Vitelli M., Mehrtash H., Assatory A., Tabtabaei S., Legge R.L., Rajabzadeh A.R. (2021). Rapid and non-destructive determination of protein and starch content in agricultural powders using near-infrared and fluorescence spectroscopy, and data fusion. Powder Technol..

[38] Li X., Zhang L., Zhang Y., Wang D., Wang X., Yu L., Zhang W., Li P. (2020). Review of NIR spectroscopy methods for nondestructive quality analysis of oilseeds and edible oils. Trends Food Sci. Technol..

[39] Rady A., Fischer J., Reeves S., Logan B., Watson N.J. (2020). The effect of light intensity, sensor height, and spectral pre-processing methods when using nir spectroscopy to identify different allergen- containing powdered foods. Sensors.

[40] Pu Y., Pérez-Marín D., O’Shea N., Garrido-Varo A. (2021). Recent advances in portable and handheld NIR spectrometers and applications in milk, cheese and dairy powders. Foods.

[41] Mishra P., Passos D. (2021). A synergistic use of chemometrics and deep learning improved the predictive performance of near-infrared spectroscopy models for dry matter prediction in mango fruit. Chemom. Intell. Lab. Syst..

[42] Mishra P., Nikzad-Langerodi R. (2020). Partial least square regression versus domain invariant partial least square regression with application to near-infrared spectroscopy of fresh fruit. Infrared Phys. Technol..

[43] Mishra P., Roger J.M., Rutledge D.N., Woltering E. (2020). Two standard-free approaches to correct for external influences on near-infrared spectra to make models widely applicable. Postharvest Biol. Technol..

